# Palliative radiotherapy for painful lymph node metastases

**DOI:** 10.1186/s13014-021-01900-8

**Published:** 2021-09-16

**Authors:** Kohsei Yamaguchi, Tetsuo Saito, Ryo Toya, Etsushi Tomitaka, Tomohiko Matsuyama, Yoshiyuki Fukugawa, Takahiro Watakabe, Hirohito Otsuka, Natsuo Oya

**Affiliations:** 1 Department of Radiation Oncology, Kumamoto University Hospital, 1-1-1 Honjo, Chuo-ku, Kumamoto, 860-8556 Japan; 2 Department of Radiation Oncology, Arao Municipal Hospital, Arao, Japan; 3 Department of Radiation Oncology, Hitoyoshi Medical Center, Hitoyoshi, Japan; 4 Department of Radiation Oncology, Kumamoto Medical Center, Kumamoto, Japan

**Keywords:** Palliative radiotherapy, Painful lymph node metastases, Pain interference, Predominance of other pain

## Abstract

**Background:**

There is limited evidence concerning radiotherapy for painful lymph node metastases (PLM). We evaluated the effectiveness of radiotherapy for PLM using the International Consensus Endpoint in a subgroup analysis of a prospective observational study.

**Methods:**

In the primary study, 302 patients received radiotherapy for painful tumors. Among them, those treated with palliative radiotherapy for PLM were analyzed in the present study. We used the Brief Pain Inventory short form to evaluate the intensity of pain and the pain interference in patient's life. We collected the Brief Pain Inventory and analgesic data at baseline and at 1, 2, and 3 months after the start of radiotherapy. Pain response was assessed using the International Consensus Endpoint. Patients were diagnosed with a predominance of other pain (POP) if non-index pain of a malignant or unknown origin was present and had a greater 'worst pain' score than the index pain.

**Results:**

Radiotherapy for PLM was performed on 25 patients. In total, 15 (60%) patients experienced a pain response. The pain response rates for evaluable patients were 66%, 67%, and 57% at 1-, 2-, and 3-month follow-ups, respectively. At baseline and at 1, 2, and 3 months, the median index pain scores were 7, 2, 0, and 0.5, respectively. At 1 month, all pain interference scores were significantly reduced from baseline. Four (16%) patients experienced POP within three months.

**Conclusion:**

Radiotherapy for PLM improved pain intensity and pain interference. Palliative radiotherapy may be a viable treatment option for PLM.

## Introduction

Radiotherapy is an important treatment option for cancer-related pain [1, 2]. The pain response rate after radiotherapy for painful various tumors was reportedly 47–80% [3–6]. Among them, radiotherapy for painful bone metastases, which has been investigated extensively [3, 6, 7], was strongly recommended by the World Health Organization [8]. However, there are few studies of palliative radiotherapy for painful tumors other than bone metastases [9]. This paucity of evidence may be, at least in part, the reason why the World Health Organisation Guidelines do not mention the use of palliative radiotherapy for painful non-bone-metastasis [8].

To the best of our knowledge, few small studies have investigated radiotherapy for painful lymph node metastases (PLM) [10, 11]; however, due to the retrospective study design, their results on its effectiveness were inconclusive. Moreover, pain response was assessed based only on the intensity of pain without considering analgesic use.

In a subgroup analysis of a prospective observational study, we evaluated the effectiveness of radiotherapy for PLM using the International Consensus Endpoint in 2012 [12].

## Materials and methods

### Patients and study design

**Fig. 1:**
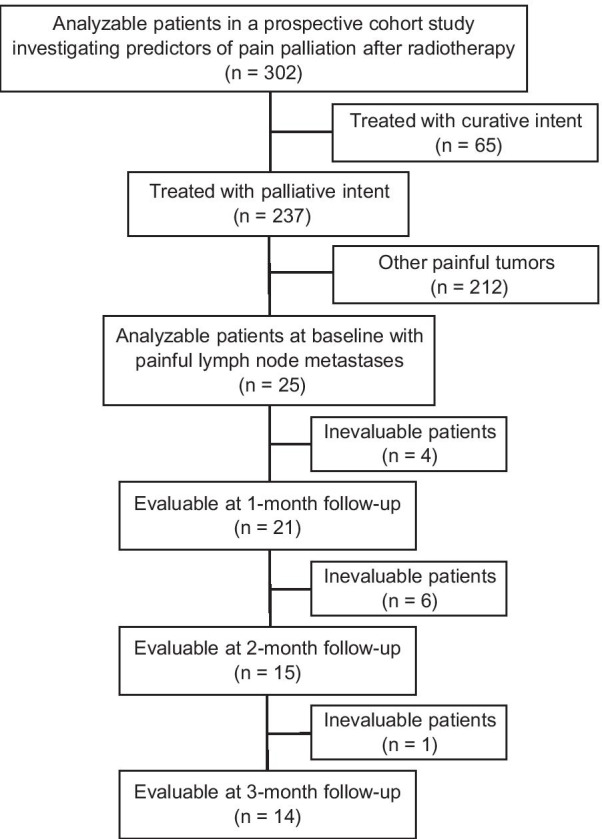
Flow diagram of the study cohort

We performed a subgroup analysis of a previously published prospective three-center observational study. In the primary study, 302 patients were scheduled to receive radiotherapy for their painful tumors. We sought to identify the predictors of pain response after radiotherapy for painful tumors [13]. Among these patients, those treated with palliative radiotherapy for PLM were analyzed in the present study (Fig. 1). Palliative radiotherapy was defined as treatment aiming to relieve pain or whose radiation field did not cover all tumors identified by diagnostic imaging [13]. Dose fractionations were determined at the discretion of the radiation oncologists. The present study was approved by the institutional review board of the participating centers. Written informed consent was obtained from all enrolled patients in the primary study.

### Evaluation

The patients were evaluated as previously reported [13]. The Brief Pain Inventory (BPI) short form was used to evaluate the intensity of pain and the pain interference in patient's life using an 11-point scale (0–10) [14]. A higher BPI score indicated more intense pain, greater disability, and poorer well-being. Patients reported the worst pain they experienced (in terms of the index pain caused by the irradiated tumor [15]) within the previous three days. The BPI assesses pain interference in seven subscales: general activity, mood, walking ability, normal work, relations with other people, sleep, and enjoyment of life [14]. BPI pain interference is typically scored as the mean of the seven interference items, and this mean can be used if more than 50%, or four out of seven items, were present on a given administration [16].

We collected the BPI and analgesic data at baseline and at 1, 2, and 3 months after the start of radiotherapy. Pain response was assessed using the International Consensus Endpoint for clinical trials in bone metastases [12]. Patients who received radiotherapy for painful tumors were categorized as responders or non-responders. Responders included patients who experienced complete and partial responses. A complete response was defined as an index pain score of 0 with no increase in the daily oral morphine equivalent dose (OMED) [12]. A partial response was defined as a reduction in pain score of ≥ 2 without an increase in OMED or reducing analgesic use by ≥ 25% without increasing the pain score. Pain progression was defined as an increase in the index pain score of ≥ 2 without reduced OMED or an increase of ≥ 25% in the OMED without a decrease in the pain score. Indeterminate response was defined as any response that did not qualify as a complete response, partial response, or pain progression.

Non-index pain was assessed in addition to the index pain. The treating radiation oncologists differentiated between index pain caused by the irradiated tumor and non-index pain, the cause of which was not treated with radiotherapy [15]. At baseline and follow-ups, the treating radiation oncologists prospectively evaluated whether the patients experienced pain other than the index pain. For the patients with non-index pain, its intensity (the worst pain within the previous three days) and origin were recorded. When more than 1 non-index pain was present, that with the greatest intensity was recorded. Non-index pain was classified as having a malignant (tumor-related) origin, unknown origin, benign origin, or treatment-related cause. Patients were diagnosed with a predominance of other pain (POP) if non-index pain of a malignant or unknown origin was present and had a more significant 'worst pain' score than the index pain at follow-ups. The intensity of the non-index pain was compared with that of the index pain at follow-up to assess the presence or absence of POP [15].

### Statistical analysis

The Wilcoxon signed-rank test was used to compare two-time points (i.e., radiotherapy initiation versus 1, 2, or 3 months after that). Two-tailed *P* values less than 0.016 were considered significant. Bonferroni correction was applied for multiplicity. Overall survival, calculated from the initiation of radiotherapy, was estimated using the Kaplan–Meier method. Statistical analyses were performed using R version 4.0.2.

## Results

### Patients

Among 302 patients analyzed in the primary study, 25 patients received radiotherapy for PLM (Fig. 1). All patients underwent three-dimensional radiotherapy; intensity-modulated radiotherapy was not performed. The baseline patient characteristics are shown in Table 1. A median total radiation dose of 30 Gy (range, 8–60 Gy) was delivered in a median of 10 fractions (range, 1–30 Fr). Eleven patients (44%) received a total radiation dose > 30 Gy. Two patients underwent a single-fraction regimen, consisting of 1 × 8 Gy. All 25 patients completed the planned radiotherapy. Three patients underwent re-irradiation to the same PLM after 3 months (6, 6, and 12 months, respectively). Among them, 2 patients received 8 Gy in 1 fraction, and the other patient received 20 Gy in 5 fractions.

**Table 1:** Baseline patient characteristics (n = 25)

Characteristic	No	%
*Age, years*		
Median	66	
Range	36–84	
*Sex*		
Female	13	52
Male	12	48
*ECOG performance status*		
0	4	16
1	10	40
2	7	28
3	4	16
4	0	0
*Interval from first tumor diagnosis to radiotherapy, months*		
Median	15	
Range	0–239	
*Primary site of the tumors*		
Lung	5	20
Gastrointestinal system	6	24
Gynecological system	6	24
Head and neck	2	8
Urogenital system	2	8
Breast	2	8
Other	2	8
*Location of the lymph node metastases (n* = *30)*		
Neck	4	13
Supraclavicular region	5	17
Axilla	2	7
Chest	4	13
Abdomen	5	17
Pelvis	8	27
Inguinal region	2	7
*Worst pain score at baseline*		
0–2	0	0
3–4	4	16
5–7	10	40
8–10	11	44
*Neuropathic component of index pain*		
No	17	68
Yes	8	32
*Non-index pain of malignant or unknown origin at baseline*		
No	23	92
Yes	2	8
*Opioid analgesic use at baseline*		
No	9	36
Yes	16	64
*Total radiation dose, Gy*		
Median	30	
Range	8–60	
≤ 10	2	8
10–20	2	8
20–30	10	40
30–40	6	24
> 40	5	20
*Concurrent systemic therapy*		
Chemotherapy	8	32
Targeted or immune-based therapy	3	12
None	14	56

ECOG, Eastern Cooperative Oncology Group

### Pain response and predominance of other pain

In total, 15 (60%) of 25 patients experienced a pain response (complete response or partial response), and four (16%) of 25 patients experienced POP within three months after the start of radiotherapy (Tables 2, 3). The pain response rates for evaluable patients were 66%, 67%, and 57% at the 1-, 2-, and 3-month follow-ups, respectively. The intention-to-treat pain response rates for all 25 patients were 56%, 40%, and 32% at the 1-, 2-, and 3-month follow-ups, respectively. At baseline and at 1, 2, and 3 months of follow-up, the median index pain scores were 7, 2, 0, and 0.5, respectively.

**Table 2:** Pain response to radiotherapy

	1-Month follow-up (n = 21)		2-Month follow-up (n = 15)		3-Month follow-up (n = 14)	
	No	%	No	%	No	%
Complete response	7	33	7	47	5	36
Partial response	7	33	3	20	3	21
Pain progression	2	10	0	0	0	0
Indeterminate response	5	24	5	33	6	43

**Table 3:** Predominance of other pain

	1-Month follow-up (n = 21)		2-Month follow-up (n = 15)		3-Month follow-up (n = 14)	
	No	%	No	%	No	%
With POP^a^	1	5	1	7	2	17
Without POP	20	95	14	93	12	83

POP, predominance of other pain

^a^Patients were diagnosed with POP if non-index pain of malignant or unknown origin was present and showed a higher pain score than the index pain

### Analgesic use

The median daily OMED at baseline and at the 1, 2, and 3 months of follow up were 15, 23, 15, and 26.5 mg, respectively.

### Brief pain inventory pain interference scores

**Fig. 2:**
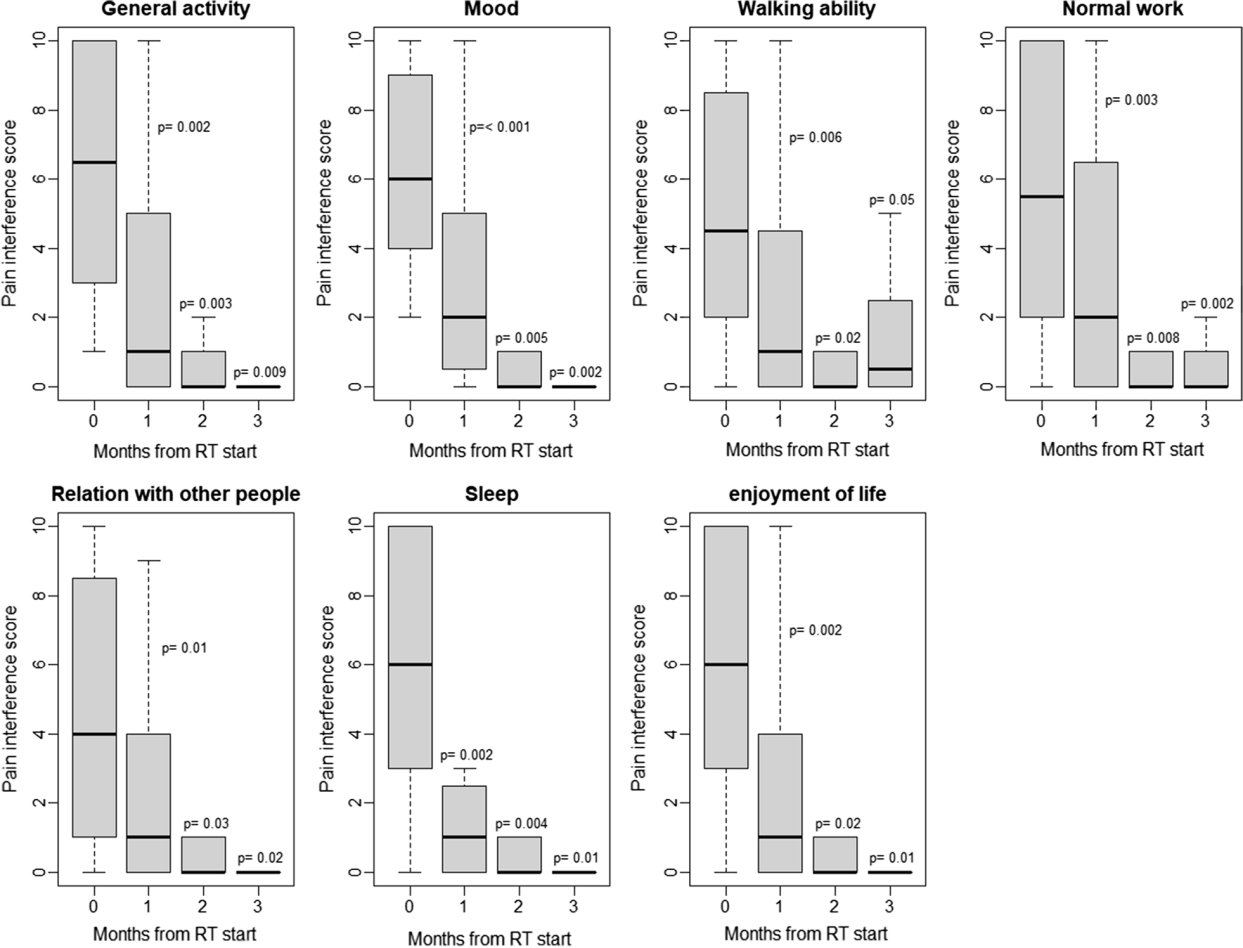
Pain interference score at baseline and at 1, 2 and 3 months of follow-up. The Wilcoxon signed-rank test was used to compare between two time points (i.e., the radiotherapy initiation versus 1, 2 or 3 months thereafter). RT, radiotherapy

**Fig. 3:**
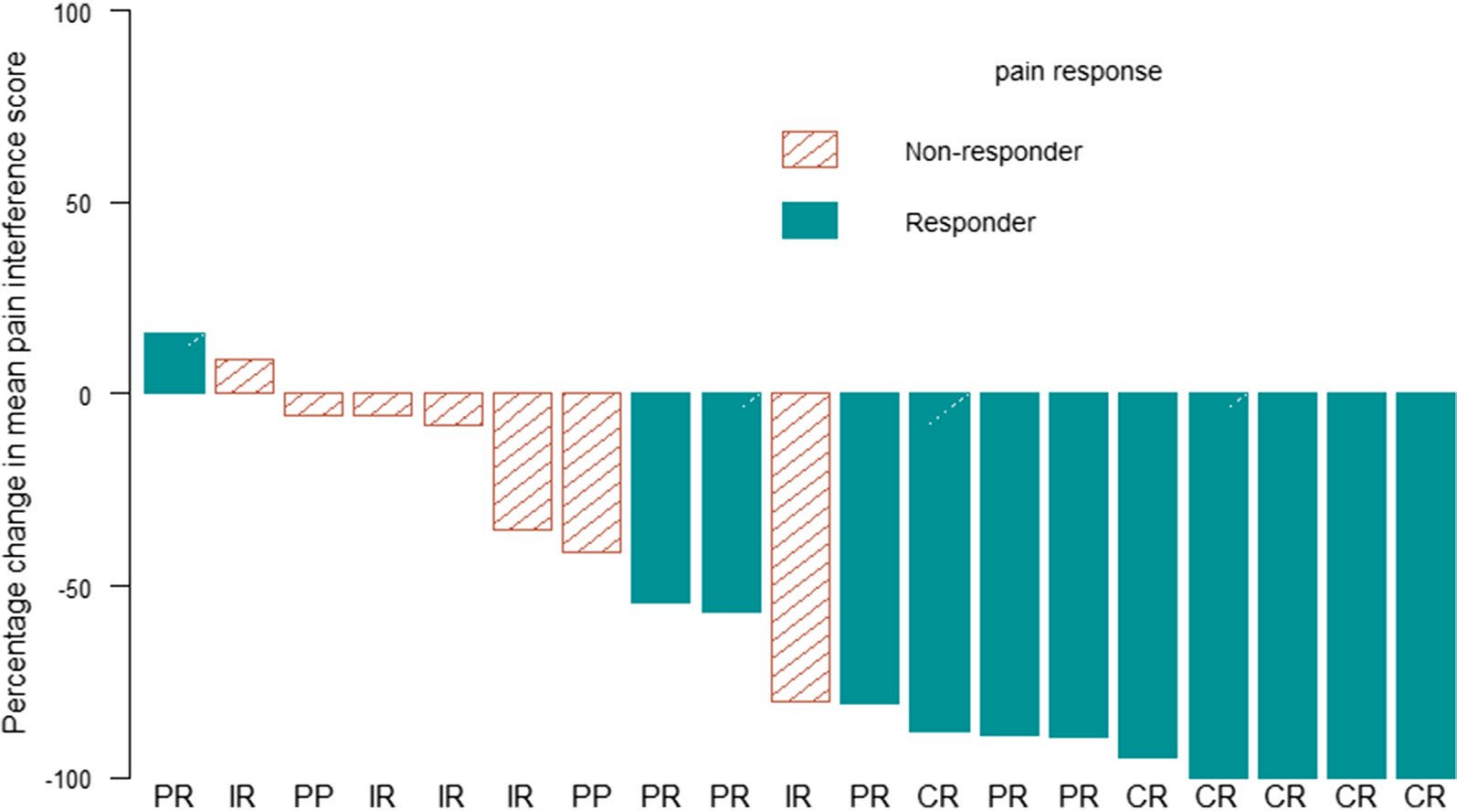
Waterfall plot of percentage change from baseline to one month follow-up in mean pain interference score. CR, complete response PR, partial response IR, indeterminate response PP, pain progression

The pain interference scores at baseline and at 1, 2, and 3 months of follow-up are shown in Fig. 2. At the 1-month follow-up, all seven pain interference scores were significantly reduced from baseline. The mean pain interference score was calculated in 19 patients, who had at least 4 available pain interference scores [16] at 1-month follow-up (Fig. 3). In 17 patients, all seven scores were available, and in the other two patients, six scores were available. Figure 3 shows the changes in the mean pain interference score per response status. Overall, responders experienced more significant reductions in pain interference than non-responders.

### Toxicity

Toxicity was evaluated based on the Common Terminology Criteria for Adverse Events version 4.0. In total, eight (32%) of the 25 patients experienced grade 1 toxicity, and four (16%) experienced grade 2 toxicity within three months after the start of radiotherapy. No patient experienced grade 3 or higher toxicity. Eight patients experienced dermatitis (grade 1, 6 patients; grade 2, 2 patients), and two patients experienced esophagitis (grade 1, 1 patient; grade 2, 1 patient). Pneumonitis (grade 2), enterocolitis (grade 1), anorexia (grade 1), pharyngeal mucositis (grade 2), and dysgeusia (grade 1) were experienced by 1 patient each.

### Survival

The median follow-up of all patients was 7.3 months. The median overall survival was 7.3 months (95% confidence interval, 2.8–11.6 months).

## Discussion

To the best of our knowledge, this was the first prospective study to analyze the effectiveness of palliative radiotherapy for PLM. We found that radiotherapy was an effective treatment for pain relief, with a pain interference reduction in PLM. As early as 1 month follow-up, all seven subscales of the pain interference were significantly reduced from baseline. POP was observed in some patients only.

There has been a few previous studies on palliative radiotherapy for PLM, with which we could compare our findings. A retrospective study that examined 68 patients receiving radiotherapy for retroperitoneal lymph node metastases reported 20 patients who presented with pain. Among them, 14 patients had extra-retroperitoneal metastases, and the other six patients had isolated retroperitoneal lymph node metastases [10]. On a 0–10 numeric rating scale, the numbers of patients in the extra-retroperitoneal metastases group with < 30%, 30–70%, and ≥ 70% pain relief were 2 (14.3%), 4 (28.6%), and 6 (42.9%), respectively. A retrospective study that examined stereotactic body radiotherapy in 22 patients with iliac lymph node metastases reported 12 patients with pain at baseline [11]. The experienced pain was not documented in terms of the pain evaluation scale. Seven patients achieved pain relief at the end of the treatment, and the other five patients reported improvement at the one-month follow-up [11]. In contrast to these studies, we assessed pain palliation based on the International Consensus Endpoint and evaluated pain interference changes using prospective data.

A systematic review of radiotherapy for painful bone metastases in prospective nonrandomized studies reported a pain response rate of 55% and a complete response rate of 15% [17]. These response rates were comparable with those of the present study (57–67%).

We previously showed that in patients with POP after radiotherapy, opioid analgesic use increased, potentially lowering the response rates [15]. In the present study, POP was only observed in a minority of the patients; this suggested that patients with PLM may benefit from local palliative therapy, such as radiotherapy.

This study had certain limitations. First, a small number of patients were assessed in this study. The sample size hampered the analysis of the predictors of pain response. Future larger studies are necessary to analyze the relation between pain response and patient/tumor/treatment characteristics, preferably by multivariable analysis. Second, the present study was post hoc, and our subgroup analysis was not a priori declared. The study findings should be confirmed in future studies. Third, the rates of attrition were relatively high, which is expected in studies investigating palliative radiotherapy [18].

## Conclusion

In the present secondary analysis of a prospective observational study, radiotherapy for PLM improved pain intensity and pain interference with mild toxicity. Palliative radiotherapy may be a viable treatment option for PLM. Larger studies are warranted to investigate the efficacy and toxicity of palliative radiotherapy for PLM.

## Data Availability

The datasets used and/or analysed during the current study are available from the corresponding author on reasonable request.

## Abbreviations

PLM: Painful lymph node metastases
POP: Predominance of other pain
BPI: Brief pain inventory
OMED: Oral morphine equivalent dose
